# Water Deficit Elicits a Transcriptional Response of Genes Governing d-pinitol Biosynthesis in Soybean (*Glycine max*)

**DOI:** 10.3390/ijms20102411

**Published:** 2019-05-15

**Authors:** Kathryn Dumschott, Julie Dechorgnat, Andrew Merchant

**Affiliations:** 1 Rheinisch-Westfälische Technische Hochschule Aachen University, 52062 Aachen, NRW, Germany; 2 The University of Sydney, Sydney NSW 2006, Australia; julie.dechorgnat@sydney.edu.au (J.D.); andrew.merchant@sydney.edu.au (A.M.)

**Keywords:** cyclitols, metabolism, gene expression, water deficit

## Abstract

d-pinitol is the most commonly accumulated sugar alcohol in the Leguminosae family and has been observed to increase significantly in response to abiotic stress. While previous studies have identified genes involved in d-pinitol synthesis, no study has investigated transcript expression in planta. The present study quantified the expression of several genes involved in d-pinitol synthesis in different plant tissues and investigated the accumulation of d-pinitol, *myo*-inositol and other metabolites in response to a progressive soil drought in soybean (*Glycine max*). Expression of *myo*-inositol 1-phosphate synthase (*INPS*), the gene responsible for the conversion of glucose-6-phosphate to *myo*-inositol-1-phosphate, was significantly up regulated in response to a water deficit for the first two sampling weeks. Expression of *myo*-inositol *O*-methyl transferase (*IMT1*), the gene responsible for the conversion of *myo*-inositol into d-ononitol was only up regulated in stems at sampling week 3. Assessment of metabolites showed significant changes in their concentration in leaves and stems. d-Pinitol concentration was significantly higher in all organs sampled from water deficit plants for all three sampling weeks. In contrast, *myo*-inositol, had significantly lower concentrations in leaf samples despite up regulation of *INPS* suggesting the transcriptionally regulated flux of carbon through the *myo*-inositol pool is important during water deficit.

## 1. Introduction

Sugar alcohols are an abundant class of molecules found in nearly all plant species. Due to their physiochemical properties, stability [1,2,3] and ability to be easily transported [4,5,6], the accumulation of sugar alcohols is often cited as an adaptive mechanism for tolerating environmental changes. Increased concentrations of sugar alcohols have been observed in response to abiotic stresses, such as water deficit and high salinity [2,7,8]. While the biosynthesis of sugar alcohols has been studied in a wide range of plant genera, the specific molecular mechanisms involved in their synthesis remain relatively understudied.

With the exception of a few well characterised pathways, such as mannitol, the molecular mechanisms controlling the synthesis and regulation of sugar alcohol accumulation and their role in whole plant metabolism remains unclear [9]. This is particularly true for the cyclitols, or cyclic sugar alcohols (for review, see [10]). To date, the most comprehensive characterisation of cyclitol biosynthesis is that of d-pinitol (3-*O*-methyl-d-*chiro*-inositol), a cyclitol that is nearly ubiquitous in the Leguminosae family. d-Pinitol is the most abundant sugar alcohol in many Leguminosae species including *Glycine max* (soybean) [1].

**Figure 1:**
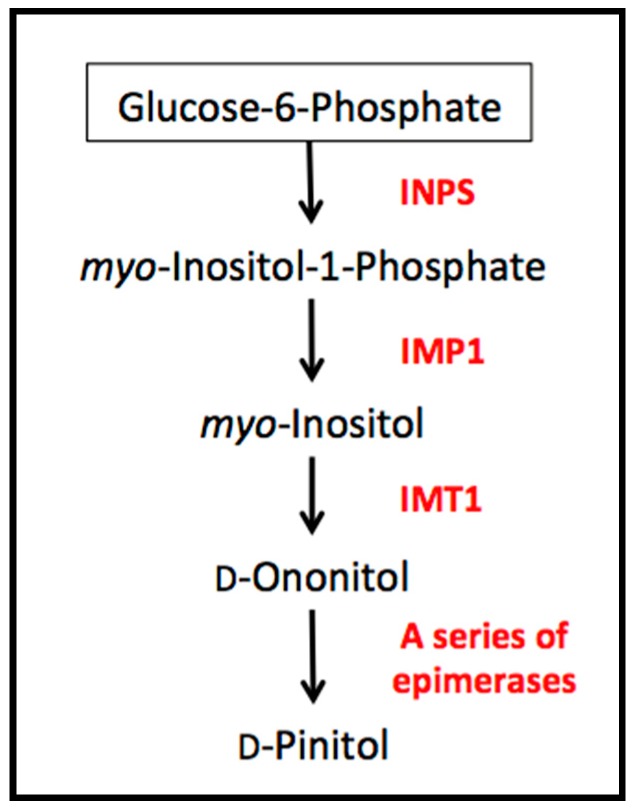
The d-pinitol biosynthetic pathway proceeds from the precursor glucose-6-phosphate. Abbreviations—INPS: *myo*-inositol-1-phosphate synthase; IMP1: inositol monophosphatase; IMT1: *myo*-inositol-*O*-methyltransferase.

The biosynthetic pathway of d-pinitol is relatively short, closely linked to substrates involved in primary metabolism (Figure 1). Occurring via only a few steps, the pathway begins with glucose-6-phosphate which is converted to d-ononitol via *myo*-inositol; the last step is the epimerization of d-ononitol into d-pinitol [1]. While d-ononitol has been reported in soybean tissues [11], it is a difficult intermediate to detect as it is rapidly converted to d-pinitol in plants capable of the final epimerization step. Past studies have focused on the isolation of specific genes in order to clarify the biosynthetic pathway of d-pinitol. For example, Ishitani et al. [12] observed that salinity stress increased *INPS* (*myo*-inositol 1-phosphate synthase) transcript in the facultative halophyte *Mesembryanthemum crystallinum* (ice plant). *INPS* is responsible of the first step in d-pinitol synthesis, the conversion of glucose-6-phosphate to *myo*-inositol-1-phosphate, indicating a diversion of carbon allocation to the sugar alcohol class of compounds.

Earlier work by Vernon et al. [13] examined the impact of the overexpression of the ice plant methyl transferase *McIMT1* in tobacco (a glycophyte). Transformed plants accumulated d-ononitol (1-d-4-*O*-methyl *myo*-inositol), a product not detectable in non-transformed plants. This study confirmed ice plant *McIMT1* encodes the *myo*-inositol *O*-methyltransferase responsible for converting *myo*-inositol into d-ononitol [13,14]. It was later shown that the synthesis of d-ononitol via IMT1 required a methyl group donated by *S*-adenosyl-L-methionine (SAM) [15]. Therefore, the IMT1 reaction is considered dependant on the activated methyl cycle to provide SAM substrate for the methylation of *myo*-inositol [16].

Combined, these studies suggest some level of transcriptional regulation of d-pinitol biosynthesis in plant tissues. However, in planta changes in transcriptional expression in response to environmental stress are largely unknown. The most well-designed example to address this gap was that of Streeter et al. [17] who compared gene expression in soybean lines varying in their ability to accumulate d-pinitol. In that study, the authors correlated higher concentrations of d-pinitol with greater IMT1 activity; supporting earlier transgenic work that IMT1 is the key regulatory step in d-pinitol biosynthesis. However, the authors also noted the developmental and spatial gradients in d-pinitol concentrations within plant tissues. d-Pinitol concentrations were found to be highest in upper leaf nodes and were observed to decrease over time in agreement with Ford [7] who also noted d-pinitol accumulated to higher concentrations in younger, more recently expanded leaves located at the top of the plant. More recently, d-pinitol concentration has been observed to vary spatially between different plant organs of fenugreek (*Trigonella foenum graecum L.*) [18].

To date, no study has investigated the quantitative gene expression profile of this pathway *in planta* in response to abiotic stress.

The prevalence of d-pinitol accumulation in a range of species coupled with the short length and proximity of its pathway to primary metabolism makes it an excellent candidate for future use as a selective trait for the improved resilience of plants to abiotic stress. However, key molecular mechanisms responsible for d-pinitol synthesis and their spatial and temporal patterns must first be elucidated. For this investigation, quantitative PCR was used to characterize expression of the genes *IMT1* and *INPS*, which encode enzymes responsible for d-pinitol biosynthesis. Expression patterns were compared to metabolite accumulation in both control and water deficit conditions. Specifically, this study aimed to address the following hypotheses: (1) a progressive soil drought will elicit the accumulation of d-pinitol in soybean; (2) metabolite accumulation will vary according to organ location in plant; (3) a quantitative increase in transcript abundance for genes governing d-pinitol biosynthesis will be observed in plants subjected to progressive soil drought; (4) transcript response will vary according to organ location in plant.

## 2. Results

### 2.1. Physiological Responses to A Progressive Soil Drought

**Figure 2:**
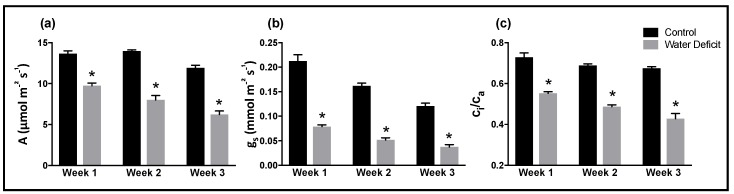
Net photosynthesis (A) (**a**), stomatal conductance (g_s_) (**b**) and the ratio of intercellular CO_2_ concentration to atmospheric CO_2_ concentration (C_i_/C_a)_ (**c**) for soybean plants subjected to drought (grey columns) and control (black columns) conditions over a period of 3 weeks. A total of 4 plants were measured every week and for each treatment. Individual plants were measured 3 times over a span of 4 h. Bars represent the standard error from the mean (*n* = 4). Asterisk (*) indicates significant difference between the drought and corresponding control column (*p* < 0.05).

Gas exchange data collected each sampling week showed significant differences between control and drought stressed plants, indicating physiological responses to the imposition soil drought (Figure 2). Net photosynthesis rate (Figure 2a) was significantly lower in drought stressed samples for each sampling week, indicating that less carbon was being assimilated into the plant system. Stomatal conductance measured from drought stressed plants was less than half of control plant conductance rates for all three sampling weeks (Figure 2b). The ratio of intercellular CO_2_ concentration (Ci) to atmospheric CO_2_ concentration (Ca), depicted as Ci/Ca, in drought stressed plants was significantly lower than in control plants for each sampling week (Figure 2c).

### 2.2. Chemical Shifts Observed within the Soluble Fraction

**Figure 3:**
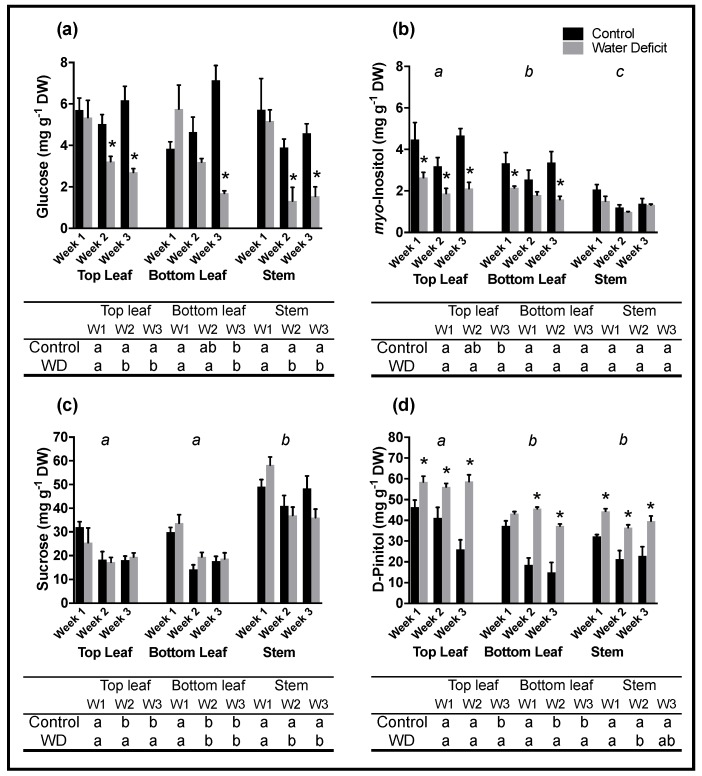
Average metabolite concentrations (mg g^−1^ DW) of glucose (**a**), *myo*-inositol (**b**), sucrose (**c**) and d-pinitol (**d**) in soybean leaf and stem samples subjected to well-watered control (black columns) and drought stress conditions (grey columns). Samples were collected at approximately 2 pm on the day of gas exchange measurements. Leaves located a node above (top leaf), a node below (bottom leaf) the gas exchange leaf, and the stem portion in between were harvested for this analysis. Columns denote the average concentration obtained from 4 plants. Bars represent the standard error from the mean (*n* = 4). Letters displayed above histogram denote significant (*p* < 0.05) differences between plant organs. Asterisk (*) indicates significant difference (*p* < 0.05) between control and treatment sample. Table below histogram denotes significant (*p* < 0.05) differences between sampling week and treatment.

Significant shifts in concentrations of the major sugar pools were observed between treatment, organ, and developmental time (Figure 3). Glucose concentrations were higher in control samples than drought stressed samples in sampling weeks 2 and 3 (Figure 3a), while week 1 concentrations were the most similar between control and drought stressed samples. No significant differences were observed in glucose concentration between different organs.

Significant changes occurred in *myo*-inositol concentrations measured in leaf samples (Figure 3b). Control top and bottom leaf samples accumulated significantly higher concentrations of *myo*-inositol than their drought stressed counterparts (*p* < 0.05). With the exception of bottom leaf drought stressed samples, which decreased over each sampling week, an initial decrease in *myo*-inositol concentration from week 1 to week 2 was observed in all other leaf and stem samples, which then increased at sampling week 3. No significant differences were observed in *myo*-inositol stem concentrations in response to drought.

Sucrose concentrations varied less than the other major sugars (Figure 3c). Overall, sucrose concentration for all plant organs sampled were highest at sampling week 1. Stem samples had significantly higher (*p* < 0.05) concentrations of sucrose compared to top and bottom leaf samples. Control stem sample concentration recovered to near original concentrations by week 3 while control leaf samples saw no real concentration changes between weeks 2 and 3. Sucrose concentrations did not vary significantly between control and drought stressed plants.

d-Pinitol concentrations were significantly higher (*p* < 0.05) in all organs subjected to drought stress than in control samples (Figure 3d). Spatially, concentrations of d-pinitol in the top leaves were significantly higher (*p* < 0.05) than concentrations found in bottom leaf and stem samples, which accumulated d-pinitol to similar concentrations. While there were no significant temporal changes in d-pinitol concentrations in drought stressed top leaves, concentrations in control plants decreased significantly (*p* < 0.05) over the three sampling weeks.

### 2.3. Transcript Responses to Water Deficit

**Figure 4:**
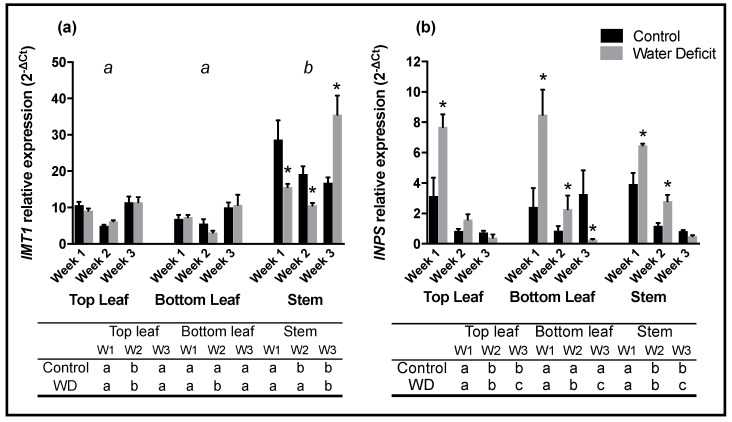
Relative expression profiles of *IMT1* (**a**) and *INPS* (**b**) genes in soybean leaf and stem samples subjected to well-watered control (black columns) and drought stressed conditions (grey columns). Samples were collected at approximately 14:00 on the day gas exchange measurements there taken. Leaves located a node above (top leaf), a node below (bottom leaf) the gas exchange leaf and the stem portion in between were harvested for this analysis. Columns denote the average concentration obtained from 4 plants. Bars represent the standard error from the mean (*n* = 4). Letters above figures denote significant (*p* < 0.05) differences between plant organs sampled. Asterisk (*) indicates significant difference (*p* < 0.05). Table below histogram denotes significant (*p* < 0.05) differences between sampling week and treatment.

Significant and varied differences in gene expression were observed between control (well-watered) and drought stressed soybean plants (Figure 4). *IMT1* gene expression varied between different plant organs (Figure 4a). Relative expression was significantly higher (*p* < 0.05) in stems than in leaves for both control (well-watered) and drought stressed plants. Neither top nor bottom leaves had significant changes in *IMT1* expression between treatments. All three plant organs sampled presented a decrease in *IMT1* expression from week 1 to week 2. Expression then increased significantly (*p* < 0.05) from week 2 to week 3 in top leaves and bottom drought stressed leaves. *IMT1* expression in stem samples displayed significant differences between treatments. Control (well-watered) samples had significantly higher *IMT1* expression in week 1 and 2 than stressed plant stems. By week 3, expression in drought stressed stems peaked to nearly twice that of the control stems. 

While *myo*-inositol phosphate synthase (*INPS*) expression was consistent between different plant organs at week 1, expression changed both temporally and in response to treatment (Figure 3b). Relative expression of *INPS* at the first week of sampling was significantly higher in drought stressed plants in all organs tested. As the weeks progressed, expression decreased significantly in water deficit until control and drought stressed expression was nearly identical for top leaves and stems and significantly lower than control expression in bottom leaves.

## 3. Discussion

The duration and intensity of the gradual drought stress imposed upon treatment plants elicited a significant response in gas exchange and metabolite accumulation, which allowed for an ideal platform to study the nature of expression patterns for genes involved in d-pinitol synthesis. Imposition of a gradual drought stress resulted in significant changes in metabolite concentrations sampled from soybean leaves and stems. This was especially pronounced for d-pinitol, which had significantly higher concentrations in drought stressed samples for all three sampling organs and for all three sampling weeks. Similar to this study, the increased accumulation of low molecular weight compounds such as sugar alcohols have often been observed in response to abiotic and osmotic stress [7,8,19], suggesting they may play an important role in osmotic adjustment and helping the plant overcome stress. The biosynthetic pathway of d-pinitol suggests that the plant is able to divert carbon away from primary metabolism into the d-pinitol pool. Higher concentrations of d-pinitol in drought stressed samples coupled with the observation that less carbon was assimilated in drought stressed plant systems (as demonstrated by significantly lower photosynthetic rates in drought stressed plants) further supports this notion. The nature of its pathway coupled with its chemical inertness [2,3] further supports the belief that d-pinitol is well suited as a stress metabolite. Future studies into d-pinitol accumulation should aim to include measurements of osmotic potential in order to clarify how significant the role of d-pinitol is with respect to osmotic adjustment. 

While the patterns of d-pinitol accumulation observed here aligned closely with observations reported in numerous previous studies, patterns of transcript abundance did not reflect the prevailing theory that *IMT1* is the most influential gene involved in the transcriptional regulation of this important pathway. Previous studies have observed varying responses in *IMT1* gene expression in response to different stress conditions. Studies involving acute stress, such as transfer of plants to a saline solution, have reported significant peaks in *IMT1* expression within one day of treatment [12,14,20] in ice plant and soybean. This response did not appear to be consistent when the plant is subject to a gradual stress as was the case in this study. One hypothesis might be that the increased production of d-pinitol during acute stress responses is achieved through the up regulation of both the *INPS* and *IMT1* transcripts. During more gradual onsets of stress exposure (as in the case of a progressive drought stress), plants may acclimate to the moderate increase of *myo*-inositol within the system and may therefore not have the need to up regulate *IMT1* expression. Under this scenario, plants must still adjust to the new growth conditions requiring both osmotic and osmo-protective compounds of which d-pinitol plays a substantial role. In this way, both the accumulation of d-pinitol and its synthesis may be beneficial under different rates of stress onset and that differing transcriptional responses may offer insight into what those roles might be.

In this study, lower rates of photosynthesis and stomatal conductance indicated that water-deficit treated plants were undergoing significant drought stress, however no significant differences in *IMT1* expression were observed in leaf samples over the three sampling weeks, a pattern in agreement with *IMT1* expression observed in ice plant [21]. Wanek et al. [8] proposed that IMT1 enzyme protein level and activity regulates d-ononitol biosynthesis and that the reaction may also be strongly dependent on concentrations of the *S*-adenosyl-methionine substrate and *myo*-inositol precursor [8]. This supports our earlier notion that the rate of stress exposure may exhibit strong influence over transcriptional responses. This also demonstrates that further studies into enzyme abundance and activity will likely clarify the significance of *IMT1* transcription and translation when the plant system is subjected to a gradual stress.

Expression of the *myo*-inositol phosphate synthase (*INPS*) gene was up regulated in drought stressed samples over the first two sampling weeks. This observation was consistent with expression responses found for *INPS* in ice plant subject to salinity stress [12]. Interestingly, despite a substantial increase in expression of the gene responsible for the production of *myo*-inositol, *myo*-inositol levels were significantly lower in stressed leaves. This has been previously reported for other legumes (for examples see [7,22]), suggesting an increased flux of carbon through the *myo*-inositol pool and into the d-pinitol pool when the plant is undergoing osmotic stress. Whilst our study did not aim to measure the flux of carbon among metabolite pools, this observation highlights the limitations to stochastic collection of plant materials, calculations of concentration by chemical analysis and subsequent conclusions regarding partitioning and allocation among competing chemical pathways. To date, no conclusive evidence has been obtained to document the metabolism of d-pinitol *in planta,* perhaps, exonerating this pathway from such limitations. On this background, we suggest that under gradual stress imposition, expression of *INPS* is the most influential gene determining the synthesis of d-pinitol accumulation under the conditions of this study.

Many other patterns of gene expression, which may have important consequences for d-pinitol accumulation in both time and space, were observed. Expression levels of target genes and metabolite concentrations varied substantially throughout the plant. *IMT1* expressions were higher in stems than in leaves while d-pinitol was found to accumulate to its highest concentrations in the top leaves of water deficit plants. This high to low concentration gradient, also observed in other plant taxa by both Streeter [17] and Ford [7], may point to the translocation of d-pinitol to the upper plant nodes in order to maintain metabolism in the younger, expanding leaves. In contrast to fenugreek [18], d-pinitol accumulation seemed to follow spatial (high to low) distribution which was not dependent on plant organs, as stem and bottom leaf samples had very comparable d-pinitol concentrations. Notable concentration gradients coupled with spatial differences in gene expression further emphasize the complexity of d-pinitol accumulation and demonstrate the necessity of future studies to be conducted on a whole plant basis in order to fully understand the adaptive process that plants undergo in response to osmotic stress.

This study, which aimed to elucidate the molecular mechanisms at play in the synthesis and accumulation of d-pinitol within soybean, found that *INPS*, and not *IMT1,* was transcriptionally up regulated in response to a gradual drought stress. Future studies into enzyme abundance and activity of *IMT1* and *INPS* as well as into spatial accumulation of d-pinitol on a whole plant basis are needed to further elucidate patterns of d-pinitol accumulation and the related gene expression profiles. This holistic understanding will help to develop d-pinitol accumulation as a selective trait for the improved resilience of plants to abiotic stress.

## 4. Materials and Methods 

### 4.1. Experimental Design

The commercially available soybean line ‘Snowy’ was selected for this study due to its widespread use in Australia. Seeds were planted in trays containing seed raising mix (Osmocote^®^, Scotts Australia Pty, Bella Vista, NSW, Australia) in a controlled environment chamber set at a day/night temperature of 25 °C/15 °C, 20% relative humidity, PAR to 350 μmol m^−2^s^−1^ and a 12 h light period. Approximately 10 days after germination, seedlings were transferred into 5 litre pots containing potting mix. Plants were watered to field capacity and allowed to grow for a period of approximately 20 days before the imposition of water deficit (resulting in a soil drought) and subsequent sampling began. In total, 24 plants were used: 12 were allocated to control treatment (watered to field capacity every day) and 12 were subjected to a water deficit (50% of water given to control plants) which was determined gravimetrically. This water deficit of 50% was selected as it induced a significant physiological response in plants subject to the deficit while also allowing for the continued fixation of carbon into the plant, ensuring the plant system would not shut down.

### 4.2. Physiological Measurements and Sample Preparation

The first day of measurements (defined as ‘week 1’) took place 9 days after the drought stress was imposed. Gas exchange and photosynthesis rates were measured using a WALZ GFS-3000 portable infra-red gas analyzer (Walz Heinz GmbH, Effeltrich, Bavaria, Germany). Cuvette temperature was set to 25 °C and PAR was set to 350 μmol m^−2^ s^−1^ to mimic the light intensity within the chamber. Gas exchange took place on a newly expanded leaf located approximately 3 nodes from the top of the plant.

Sampling began at approximately 09:00 and continued until 13:00. For sampling, 4 control and 4 treatment plants were measured for three rounds, with two readings being logged per plant per round. The second and third sampling days (defined as ‘week 2’ and ‘week 3’) occurred 16 and 22 days after the imposition of the water deficit respectively. 

Once gas exchange measurements were completed, leaf and stem samples were snap-frozen in liquid nitrogen and stored at −80 °C until further use. For each plant, top leaf, bottom leaf and stem samples were taken. For the purpose of this study, these were defined as follows:

- Top leaf: sample harvested from the first node directly above the node containing the WALZ sampling leaf.
- Bottom leaf: sample harvested from the first node directly below the node containing the WALZ sampling leaf.
- Stem: sample harvested from the section located between the top and bottom leaf nodes.

Leaf samples were ground frozen using the 2010 Geno/Grinder^®^ (SPEX^®^ SamplePrep, Metuchen, NJ, USA). Stem material, too tough for the grinder, were ground by hand using a mortar and pestle. Samples were then stored at −80 °C until further use.

### 4.3. Chemical Analysis

A small amount of frozen ground sample was transferred to a labelled 2 mL microcentrifuge tube, microwaved for approximately 45 s to ensure metabolism was stopped (according to [23]) and left in a 70 °C oven to dry overnight. Once dried, between 30 and 50 mg of sample was transferred into a screw cap vial and hot water extractions were performed according to Merchant et al. [24] using 0.1% pentaerythritol (98+%, *Alfa Aesar*, Haverhill, MA, USA) as an internal standard. Extracted samples were then stored at −80 °C until they were analysed on a gas chromatograph triple quadrupole mass spectrometer (GC-QQQ). 

In order to analyse non-polar analytes, samples were first derivatised according to Merchant et al. [25]. The separation and quantification of target metabolites was completed using an Agilent 6890A gas chromatograph with QQQ 7000 mass selective detector on scan mode from 50–500 AMU (70 eV) (Agilent Technologies, Santa Clara, CA, USA) according to the protocol detailed in Merchant et al. [26]. Peaks were integrated and compound data was extracted using Agilent MassHunter Workstation software (Agilent Technologies, Santa Clara, CA, USA). Scanned data for a mixed standard was extracted to determine the most abundant or best-extracted ion peak for each compound (See Table 1). Despite its importance as an intermediate in the d-pinitol pathway, d-ononitol could not be quantified in the samples. Metabolite concentrations are reported as mg g^−1^ dry weight (DW) sample material.

### 4.4. RNA Extraction and cDNA Preparation

RNA was extracted from leaf and stem samples using a ZR Plant RNA MiniPrep™ kit (Zymo Research, Irvine, CA, USA). RNA samples were quantified on a NanoDrop Lite Spectrophotometer (Thermo Scientific™, Waltham, MA, USA) and 1 µg RNA of each sample was treated with DNaseI Amplification Grade (Sigma-Aldrich, St. Louis, MO, USA) to remove genomic DNA contained in the sample. After a heat step to deactivate the DNaseI (70 °C for 10 min in the presence of 50 mM EDTA to prevent denaturation), samples were immediately reverse transcribed to cDNA using iScript^TM^ cDNA Synthesis Kit (BioRad Laboratories, Hercules, CA, USA).

To test that the cDNA reaction was successful and to confirm that no genomic DNA remained in the samples, a test PCR was performed using primers designed over an intron. PCR reactions were set up using MyTaq^TM^ DNA Polymerase (Bioline, Alexandria, NSW, Australia) and were run in a BioRad T100^TM^ Thermal Cycler (Bio-Rad Laboratories, Hercules, CA, USA) with the following protocol: 95 °C for 1 min, followed by 35 cycles of a denaturing step of 95 °C for 15 s, an annealing step of 51 °C for 15 s, and an extension step of 72 °C for 1 min. The protocol finished with a final extension step of 72 °C for 10 min. Reactions were run on a 2% agarose gel and imaged with a Chemidoc XRS+ with Image Lab™ software (Bio-Rad Laboratories, Hercules, CA, USA) to visualize PCR products.

### 4.5. Primer Design

Primers for quantitative PCR were designed for target and reference genes (Table 2). Target genes were *IMT1* and *INPS*. *IMT1* primers were found in Wang et al. [20] and *INPS* primers were designed using NCBI primer 3 and BLAST software. Reference genes, *SKIP16, Fbox* and *UNK2* were selected from [27], [28], and [29] respectively. All reference genes were chosen for their stability under drought treatment. All primer sets were tested to confirm that they yielded one PCR product consistent with the target base pair size. 

### 4.6. Quantitative PCR

Primer efficiencies and specificity were tested on all cDNA samples using SsoAdvanced™ Universal SYBR^®^ Green Supermix (Bio-Rad Laboratories, Hercules, CA, USA). Reactions were pipetted into a 96 well PCR plate (Bio-Rad Laboratories, Hercules, CA, USA) and run on an Agilent Mx3005P QPCR System (Agilent Technologies, Santa Clara, CA, USA) with the following protocol: a polymerase activation and DNA deactivation step of 95 °C for 30 s followed by 40 cycles of 95 °C for 10 s and an annealing step of 60 °C for 30 s. The protocol finished with a dissociation curve with a 60 °C starting temperature. All primers used in the study presented efficiencies between 92.1% and 102.5% (well within the target range of 90% to 110%) and a dissociation curve containing one peak and no peak in the no template control, indicating good primer specificity. 

Expression of the target genes was expressed relative to the reference genes in order to normalize cDNA concentrations between samples. First, the difference between the cycle threshold (C_t_) of the target gene and the average of the C_t_ of the three reference genes for the same sample was calculated according to:

ΔC_t_ = C_t_ of target gene−AVERAGE (C_t_ of reference genes) (1)

The relative expression of the target gene was then calculated as follows:

Relative expression = 2^(−ΔCt)^ (2)

### 4.7. Statistical Analysis

A restricted maximum likelihood (REML) analysis was run on GenStat 15th Edition (VSN International, Hemel Hempstead, UK) to determine if a significant temporal effect on gas exchange, metabolite concentration, and gene expression had occurred. A second REML analysis was then run on GenStat 15th Edition (VSN International, Hemel Hempstead, UK) to determine if a significant treatment effect on gas exchange, metabolite concentration, and gene expression had occurred. A least significant difference (LSD) test was then done to determine similar groups and see where significant temporal and treatment differences had occurred.

## Figures and Tables

**Table 1:** Information used to identify target compounds for metabolite analysis.

Compound Name	Precursor Ion	Retention Time min^−1^	Ion Polarity
Sucrose	217.0	28.8	Positive
Glucose	203.9	18.8	Positive
*myo*-Inositol	304.9	20.3	Positive
d-Pinitol	259.9	17.3	Positive
Pentaerythritol	190.9	13.5	Positive

**Table 2:** Primers designed to isolate target genes involved in d-pinitol synthesis and reference genes used to normalize samples in soybean leaves and stems.

Gene (Symbol)	Forward Sequence (5′-3′)	Reverse Sequence (5′-3′)	BP
**Target Genes**			
d-*myo*-Inositol-3-Phosphate Synthase (*INPS*)	GTTGTACTGTGGACTGCCAA	GGCATACAAGGTGGAAGGAG	129
*myo*-Inositol *O*-Methyltransferase (*IMT1*)	GGCACTACCAGACAATGGGAAG	ACAGCAAACAACTCGGAAACC	202
**Reference Genes**			
SKP1/ASK-Interacting Protein 16 (*SKIP16*)	GAGCCCAAGACATTGCGAGAG	CGGAAGCGGAAGAACTGAACC	60
Fbox Protein (*FBOX*)	AGATAGGGAAATTGTGCAGGT	CTAATGGCAATTGCAGCTCTC	93
UNK2 (*UNK2*)	TGTGCTCTGTGAAGAGATTG	TCATAATCTGTGTGCAGTTC	156
